# Preconception Care Uptake and Immediate Outcomes among Discordant Couples Accessing Routine HIV Care in Kenya

**DOI:** 10.1155/2020/1675987

**Published:** 2020-06-09

**Authors:** Nyawira Gitahi, Sheila Juliet Eshiwani, Kenneth Mutai, Jared Ongechi Mecha, James Njogu Kiarie

**Affiliations:** ^1^Institute of Tropical Medicine and Infectious Disease (ITROMID), Kenya Medical Research Institute, Nairobi, Kenya; ^2^Department of Clinical Medicine and Therapeutics, University of Nairobi, Nairobi, Kenya; ^3^Kenyatta National Hospital, Nairobi, Kenya; ^4^Department of Obstetrics and Gynaecology, University of Nairobi, Nairobi, Kenya

## Abstract

**Introduction:**

A large proportion of new HIV infections occur within discordant partnerships making discordance a significant contributor to new HIV infections in Africa. Despite the role of preconception care for HIV discordant couples, there is little data on fertility desire and preconception care uptake. This study aimed at documenting fertility desire (desire to conceive), determining the uptake of preconception care, identifying HIV prevention strategies used during preconception care, and determining immediate conception outcomes among HIV discordant couples in Kenya.

**Methods:**

We retrospectively extracted electronic medical record data on discordant couples at an HIV care discordant couples' clinic. We included data on couples who expressed a desire to conceive and were offered preconception care and followed up for 29 months. We collected data on sociodemographic characteristics, preconception prevention methods, and associated outcomes.

**Results:**

Among couples, with male HIV-positive partners, there was a twofold likelihood of accepting preconception services (OR = 2.3, CI 95% (1, 1, 5.0)). A shorter discordant union was independently associated with the uptake of preconception services (OR = 0.92, CI 95% (0.86, 0.98)). The most used prevention intervention (38.5%) among discordant couples was a combination of pre-exposure prophylaxis (PrEP) by the uninfected partner, alongside HAART by the partner living with HIV. Pregnancy rates did not significantly (*p*  =  0.06) differ among those who took up preconception care versus those who did not. HIV-negative partners of couples who declined preconception care had a significantly (*p*  =  0.04) higher attrition from clinic follow-up. One confirmed seroconversion occurred; an HIV incidence rate of 0.19 per 100 person-years.

**Conclusion:**

The study demonstrates the feasibility of implementing safe and effective preconception servicesas part of routine HIV care for discordant couples living in low resource settings. The provision and the utilisation of safer conception services may be hindered by the poor retention to follow-up and care of HIV-negative partners. This challenge may impede the expected benefits of preconception care as an HIV prevention intervention.

## 1. Introduction

In high HIV prevalence settings, up to 50% of the new infections occur within HIV discordant partnerships [1–3]. HIV serodiscordant couples desiring children can knowingly risk HIV transmission with unprotected intercourse [1, 2, 4, 5]. In this regard, preconception care among discordant couples is urgent and critical both in the prevention of HIV transmission to the uninfected partner and also in the prevention of vertical transmission from mother to baby [6].

Among persons living with HIV (PLWHIV), the goals of preconception care include facilitating reproductive decision-making and minimising risks of horizontal and vertical HIV transmission [6, 7]. There are existing recommended prevention interventions such as highly active antiretroviral therapy (HAART) for all positive partners, preconception counselling, condom provision, and emphasis on planned pregnancies towards safer conception among discordant couples [6]. Additionally, the use of pre-exposure prophylaxis, artificial vaginal insemination, timed unprotected intercourse, and sperm wash have been described in the literature [8–12]. The recommended World Health Organization (WHO) preconception care package for all couples [6] includes among others, vaccination against preventable diseases, assessment for sexually transmitted infections (STIs), and nutritional and genetic assessment. There is existing data that PLWHIV who are adherent to highly active antiretroviral therapy (HAART) and maintaining an undetectable viral load have effectively no risk of sexually transmitting HIV [7, 13–15]. A 96% reduction in HIV transmission among discordant couples [13] even in the presence of inconsistent condom use [16] and over long periods (up to 10 years) has been reported. Moreover, maternal viral suppression to undetectable levels before conception, during pregnancy, and at the time of delivery makes perinatal HIV transmission almost entirely preventable [17, 18]. Given these apparent benefits, the WHO now recommends preconception care as a standard component of primary care for HIV discordant couples [6].

The uptake of safer conception service in high-burden, resource-limited settings remains mostly unknown [10]. In Kenya, 50% of persons with known HIV status are in discordant partnerships [19]. Globally and in sub-Saharan Africa, most of the data available on HIV prevention and conception are primarily from research settings [2, 3, 15, 17, 20]. Specifically, data gaps exist on the feasibility of the implementation of safer conception models in low- and middle-income settings [15, 21, 22].

We report on fertility desire (desire to conceive) among HIV discordant couples, uptake of preconception care, HIV prevention preferred methods, and immediate conception outcomes and couple retention outcomes among HIV discordant couples in sub-Saharan Africa.

## 2. Methods

### 2.1. Study Design

This study utilises data from a cohort of HIV discordant couples enrolled in care from April 2013 to September 2015.

### 2.2. Study Setting

We conducted this study in a public referral and teaching hospital that offers free HIV care within a clinic that caters exclusively to discordant couples. Enrolment criteria to the discordant clinic were HIV serodiscordant, age above 18 years, willingness to receive care as a couple, discordant relationship existent for at least three months, and residing within proximity to the clinic or able to attend three monthly visits.

Within the discordant couples' clinic, interventions directly targeting discordant couples were provided as per the 2015 WHO guidelines; HAART to all HIV positive partners and routine viral load monitoring [14]. Services offered to the HIV-negative partner include quarterly HIV testing, STI screening and treatment, condom and lubricant provision, and risk reduction counselling [4]. All couples who reported that they did not desire to conceive in the next six months were provided with contraception counselling and offered or referred for the contraception option of choice. We explored fertility intention as part of standard care of treatment within the clinic, usually at every visit through the question “Do you plan to conceive within the next three months?” We used routinely collected data within the electronic medical patient encounter forms.

### 2.3. Preconception-Specific Services

Couples who expressed a desire to conceive and where the HIV-positive person had achieved viral suppression were enrolled to preconception care sessions. These scheduled monthly sessions, included discussions on safer conception practices and the importance of optimum viral suppression (<1000 copies/ml). Preconception interventions discussed were PrEP and timed unprotected intercourse (TUI) during ovulation. Clinic staff also demonstrated self-artificial vaginal insemination (AVI). The preconception care clinic team consisted of a general practitioner medical doctor, a nurse trained in reproductive health care, and a gynaecologist. Preconception care required the couple to attend the clinic every month until conception occurred for at least one year. Failure to conceive after one year of follow-up resulted in a referral for gynaecological management and further clinical evaluation.

We extracted data for this study from routinely collected electronic medical records during the provision of care discordant couples' clinic between April 2013 and September 2015. In this study, we only analysed data from couples enrolled for HIV care at the discordant couples' clinic who expressed a desire to conceive during routine visits included analysis.

We obtained ethical approval from the Kenyatta National Hospital/University of Nairobi joint Ethical and Scientific Committee for the use of routine programmatic data. The outcome measures were uptake of preconception care services, uptake of HIV prevention methods, conception outcomes, and discordant couple retention to care. We also examined sociodemographic characteristics and seroconversion events.

Data were analysed using SPPS version 23, IBM Corporation, Armonk, New York. Means and standard deviations (SD), medians and interquartile ranges (IQR), and proportions summarised the continuous and categorical variables, respectively. Chi-square and Mann–Whitney *U* tests were used for comparisons between those who took up preconception care versus those who did not and to compare those who conceived versus those who did not. Multiple logistic regression models were used to compute odds ratios using age, sex, duration of union, and use of ART as covariates of uptake of preconception care. Statistical significance was set at 95% (*p* value < 0.05 ≤ 0.05).

## 3. Results

**Figure 1:**
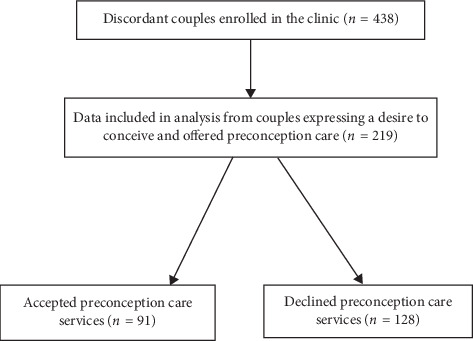
Flow diagram indicating sample of discordant couples' data included in the analysis, 133 for the study.

Out of a total of 438 HIV serodiscordant couples enrolled in the discordant couples' clinic, we included 219 (50%) who expressed a desire to conceive in the analysis. Out of the 219 who expressed a desire to conceive only 91 (41.6%) of couples took up preconception care services (Figure 1).

### 3.1. Characteristics of the HIV Discordant Couples and Factors Associated with Preconception Care Uptake

There was no significant difference in the comparison of the mean ages of males (*p* = 0.827) and females (*p* = 0.712) who took up preconception services compared to those who did not. Discordant couples who expressed a desire to conceive but did not take up the preconception services had a significantly (*p* = 0.015) larger proportion of female HIV-positive partners. The median duration of the union/relationship was significantly (*p* = 0.005) longer among couples who chose not to take up the preconception services. The proportion of positive partners initiated on HAART was significantly (*p* = 0.043) higher (100% compared to 95.3%) among the partners in the preconception care group. Factors independently associated with uptake of preconception care were male sex of the HIV-positive partner union (OR = 2.3, CI 95% (1, 1, 5.0)) and having a shorter duration of the discordant union (OR = 0.92, CI 95% (0.86, 0.98)) (Table 1).

### 3.2. Use of HIV Prevention Methods among Couples Receiving Preconception Care

**Figure 2:**
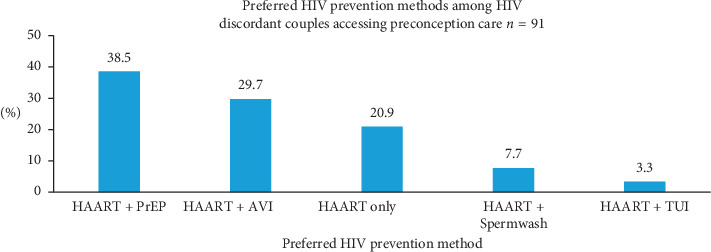
Discordant couple preferences for HIV prevention methods during preconception care.

All HIV-positive partners within the discordant couples' clinic received HAART. The prevention strategy with the highest uptake among the couples was the use of PrEP alongside HAART at 38.5%, followed by HAART and AVI at 29.7%. Within discordant unions where the HIV-infected partners were females, the use of PrEP was more common (64%) compared to couples with male HIV-positive partners (55.3%). The use of HAART only was reported among 20.9% of the couples. An interesting finding was a request for a referral for sperm wash services by approximately 8% of the couples where the male was HIV positive. These requests occurred despite couples receiving counselling on the reduced chances of transmission with optimal use of HAART and a confirmed viral load suppression (<1000 copies/ml as per national guidelines) among the HIV-positive males. We referred these couples for sperm wash services provided at private clinics at the couples' cost. The smallest proportion (3.3%) preferred timed unprotected intercourse (TUI) ( Figure 2).

### 3.3. Preconception Care Outcomes

Twenty seven (29.7%) women accessing the preconception care conceived compared to 24 (18.8%) who declined. We reported no seroconversions within the preconception care group. One uninfected partner seroconverted within the group that did not take up the services resulting in an HIV incidence rate of 0.19 per 100 person-years of follow-up. The overall couple retention was significantly (*p*=0.004) higher among couples who accepted the services. Additionally, only three (2.3%) partners in the preconception care cohort were lost to follow-up from the discordant couple care clinic compared with the 27 individuals (21%) who declined preconception care (Table 2).

## 4. Discussion

We found an unexpected low uptake of preconception care services among couples who had expressed a desire to conceive. Among couples who took up the services, we reported a clear association between the duration of the union and increased service uptake. Our study also found that discordant couples with male HIV-positive partners were more likely to take up the services. An identified gap was the high attrition from discordant clinic follow-up among the HIV-negative partners.

The low uptake of preconception care services remains unexplained. However, HIV-related stigma, fear of unfamiliar medical procedures, and lack of information about the HIV prevention interventions among community members are barriers to uptake [10]. An insightful result from this study was that there was increased uptake of preconception care services among couples where males were the HIV-infected partners. We postulate that these two findings may be related to health information gaps and relationship power dynamics. The dominant role of the male partners' fertility desire in steering couples' fertility decision-making as is often seen in the pate societies, which may have been a contributing factor to driving the uptake [22].

There may have been inadequate health education on the benefits of preconception care during the introduction of preconception care to the discordant couples attending the clinic, leading to the overall poor uptake. However, conversely, couples may also have had prior knowledge of the higher risk of receptive penile-vaginal versus incentive penile-vaginal sexual intercourse [15, 22]. This knowledge could also have contributed to lower uptake of the services mainly, where males were the HIV-negative partners as seen in this study. This finding if further interrogated would guide programmes on leveraging on male participation to improve uptake of preconception care.

The finding of the shorter duration of a discordant union as an independent determinant of increased uptake of preconception care services by the relatively young discordant couples was an exciting finding that is also demonstrated by other studies [20]. Additionally, the pregnancy rate of 9.6% in this study was within the range (9.6–16.6) reported in clinical trials which focussed on relatively young discordant couples [17, 21]. These two findings further support existing literature that indicates that a desire for children in young couples (as evidenced by median age and short duration of the union) may be a significant potential contributor to HIV transmission [10, 21]. Though the difference in pregnancy rates between those who took up the services at (29.7%) versus those who did not (18.8%) was higher but not statistically different, information provided to couples who took up the services on fertility cycles and timing of ovulation most likely contributed to the relatively higher conception rates in this cohort.

With only one reported seroconversion event, the HIV incidence rate was lower than that reported among studies in Africa, in which the HIV incidence varied between 1.2 and 22 per 100 person-years with a median of 11.1 per 100 person-years [6]. However, we note that HIV incidence may be underreported in this study, taking into account the significant loss to follow-up reported among the couples who declined preconception. Nevertheless, this study demonstrates a decrease in HIV acquisition as a result of HAART, comparable to results from clinical trials among discordant couples in real-life program implementation [13, 16, 17].

A limitation of this study was the use of retrospectively collected data which did not allow the inclusion of additional exposure variables that could have contributed to the outcomes. The study population may also be unique as a significant proportion had participated in clinical studies or expressed interest to attend discordant couples care clinics. Data on pregnancy outcomes of this cohort were challenging to obtain because once pregnancy is confirmed, they are referred to antenatal clinic and consequently, a high-risk clinic that follows up mothers and HIV-exposed infants.

This study, however, has the overarching strength of documenting the feasibility and successful implementation of preconception care interventions among discordant couples in the context of routine care in a low resource setting, providing new information outside the setting of the numerous research settings previously reported which focussed on the discordant couple.

## 5. Conclusion

We have demonstrated that in the context of routine HIV care and treatment for all couples, providing preconception care for discordant couples that express a desire to conceive is feasible and valuable. The introduction of preconception services should have robust health education and HIV prevention literacy component in order to improve couples understanding of the potential benefits. Strategies are required to mitigate the low HIV-infected partner retention enabling the actualisation of the full gains of preconception care. The introduction of conception care within discordant couple's routine care is essential and in future will play a significant role in the aversion of horizontal and vertical HIV transmission.

## Figures and Tables

**Table 1:** Characteristics of couples expressing a desire to conceive who accepted preconception care services versus those who did not.

Variable	Preconception care services		*P* value	Adjusted odds ratio
	Accepted (*n* = 91)	Not accepted (*n* = 128)		95% CI
Mean age in years of the female partner (SD)	34.1 (5.8)	34.5 (7.5)	0.712	1.00 (0.93–1.0)
Mean age in years of the male partner (SD)	39.7 (7.9)	39.5 (8.5)	0.827	1.03 (0.97–1.09)
Sex of the positive partner, *n* (%)				
Female	44 (48.4)	83 (64.8)		Ref
Male	47 (51.6)	45 (35.2)	0.015	2.3 (1.1–5.0)
Median duration of union in years (IQR)	7 (4–10)	10 (5–15)	0.005	0.92 (0.86–0.98)
HIV-positive partner on HAART, *n* (%)	91 (100.0)	122 (95.3)	0.043	—

**Table 2:** Preconception care outcomes.

Variable	Preconception care services		*P* value
Preconception care outcome, *n* (%)	Accepted (*n* = 91)	Not accepted (*n* = 128)	
Pregnancy	27 (29.7)	24 (18.8)	0.060
No	64 (70.3)	104 (81.3)	
Retention to discordant couple care, *n* (%)			
HIV-positive partner			
Active	90 (98.9)	117 (91.4)	206
Loss to follow-up	0	3 (2.3)	0.091
Dead	0	3 (2.3)	
Transfer out to other facility	1 (1.1%)	5 (3.9)	
HIV-negative partner			
Active	83 (91.2)	96 (75.0)	
Loss to follow-up	8 (8.8)	27 (21.1)	
Dead	0	0	0.004
Transfer out to other facility	0	5 (3.9)	

## Data Availability

The data used to support the findings of this study are included in the supplementary information file.

## Supplementary Materials

Supplementary Materials Table 3 Study database.
